# Activation of the maternal caregiving system by childhood fever – a qualitative study of the experiences made by mothers with a German or a Turkish background in the care of their children

**DOI:** 10.1186/1471-2296-14-35

**Published:** 2013-03-18

**Authors:** Thorsten Langer, Miriam Pfeifer, Aynur Soenmez, Vera Kalitzkus, Stefan Wilm, Wilfried Schnepp

**Affiliations:** 1 Children’s Hospital, HELIOS Centre Wuppertal, Witten/Herdecke University, Wuppertal, Germany; 2 Institute of General Practice and Family Medicine, Witten/Herdecke University, Witten, Germany; 3 Department of Nursing Science, Witten/Herdecke University, Witten, Germany

**Keywords:** Childhood fever, Fever phobia, Caregiving system, Culture, Parental experience, Qualitative methods

## Abstract

**Background:**

Childhood fever represents a frequent cause to consult a primary care physician. “Fever phobia” describes a fearful and irrational view of fever shared by many parents with different cultural backgrounds. The study aims to explain the experiences of mothers of children having a fever and to analyze the role of the mothers’ cultural background with regard to their experiences by comparing the accounts of mothers with a German with those from a Turkish background. Disease and context specific knowledge about the influence of culture can be important for effective counselling.

**Methods:**

We applied a qualitative approach using in-depth interviews with 11 mothers with a Turkish and 9 with a German background living in Germany. The interviews were conducted at the participants´ homes from May to October 2008. Data was audio-recorded and transcribed verbatim. Grounded Theory was used as a framing methodology including open, axial and selective coding. Analysis was performed in a group with members of different professional and cultural backgrounds.

**Results:**

Mothers experienced their child’s fever not merely as elevated temperature but as a potentially dangerous event. A deeply rooted urge to protect the child from harm was central to all participants’ experience. The caregiving system model offers a good theoretical foundation to explain the findings as it incorporates the unique relational quality of care giving mothers to their children. The cultural background represents an important context variable influencing the explanatory models and strategies of dealing with fever. The identified culturally influenced concepts sometimes match and sometimes conflict with medical knowledge.

**Conclusion:**

By applying the caregiving system model which is a part of attachment theory (Bowlby) maternal actions can be understood as an understandable attempt to protect the child from harm. The mothers´ decisions what to do when a child has a fever can be culturally influenced. This may lead either to a frequent use of services or to an underestimation of the child’s state of health. The mothers´ caring role and emotional state should be acknowledged; her concerns, explanatory models and strategies should be elicited and taken seriously in order to maintain a trustful relationship, provide effective counselling and thereby insuring optimal care for the children.

## Background

### Parents’ experience of childhood fever

Fever is a common condition in children and one of the most common reasons to consult a primary care physician accounting for 6-30% of all practice visits [1-3]. From a medical perspective, the underlying condition is often a harmless and self-limiting viral infection. However, in some cases fever is a response to a severe bacterial infection requiring swift and appropriate medical treatment [2].

Considering the gap between the intensive use of professional services by parents and the predominantly harmless nature of childhood fever, a number of studies have been conducted to study parents’ illness concepts. In 1980 Schmitt introduced the term “fever phobia” to describe a fearful view of fever shared by many parents [4]. The author concluded that the great concern of parents about fever is not justified medically. This first study was followed by several others identifying factors augmenting parents’ level of concern: high temperature [5,6], low educational status [7-10], single-child family [8], ethnic background [7,10-13], febrile episodes in the past [14] and the perceived degree of control in relation to the perceived threat of an illness [15,16]. Crocetti et al. repeated Schmitt’s study 20 years later and found that “fever phobia persists” [14]. The authors conclude that “pediatric health care providers have a unique opportunity to make an impact on parental understanding of fever”. The majority of studies conducted so far focus on parents’ knowledge of fever and their concerns. These have shown that parents are often not correctly informed about the temperature defining fever as a medical term [7,8,17-19], that they are unrealistically worried of permanent damage caused by fever [4,7,14,20-22], and that they administer antipyretics incorrectly [5,7,8,17-19]. However, while these studies shed light on the potential information needs of parents they fall short of explaining parents’ concerns and help-seeking behaviour. Walsh et al. tested a conceptual model of parents’ intentions to reduce their child’s fever by applying the theory of planned behaviour. It could be shown that parents’ intentions to reduce fever with medications involve a complex interplay of attitudes and subjective norms. Further, the child’s behaviour when taking antipyretic drugs itself reinforced a positive attitude towards reducing temperature [23].

Although these studies provide important insights into parental fever management, we argue that an approach that merely examines parents’ knowledge and anticipated complications of fever may not adequately represent the view of caregiving adults. To move beyond parents’ knowledge of fever and to gain a better understanding of their experiences it seems necessary to address and explore their subjective views in their roles as caregivers. We therefore address the *experience* of mothers whose child has a fever.

### Influence of the cultural background on the experience of fever

Taveras et al. showed that the cultural background influences a parent’s fever concept in a multiethnic sample in the United States [7]. For example, parents with a Latino background were more likely to believe in Latino folk illnesses, e.g. *mal de ocho,* a belief that fever can be caused by a gaze with negative intentions. Further cross-sectional studies showed differences e.g. in temperature reducing techniques between participant of different ethnic origin [12,13]. In a cross-sectional study among mothers with a German and Turkish background the authors found that the latter were more likely to give acetaminophen before the recommended interval of six hours and visited out-of-hours services more frequently [11].

Citizens with a Turkish background represent statistically the largest immigrant group in Germany. During the 1960s the federal government of Western Germany recruited more than 500 000 Turkish predominantly male working migrants as the country was experiencing rapid economic growth while simultaneously suffering from labour shortages. After the cancellation of the labour recruitment agreement in 1973 many of the so called “guest workers” decided to bring in their family members and stay in Germany permanently [24]. In 2010, 3% of the German population (2 485 000 citizens) has a Turkish background [25]. In contrast to the ethnically German re-patriates who immigrated from Central and Eastern Europe in large numbers and represent the second largest immigrant group the citizens with a Turkish background often share the same language as well as ethnic and religious (Muslim) characteristics that are different from the ethnically German and predominately Christian majority. Assuming that the “cultural distance” is larger than to the re-patriates we decided to compare the accounts of mothers with a German and a Turkish background.

The aim of this study was to explore and explain the experiences of mothers with German or Turkish backgrounds when their child has a fever. It focuses on the specific meaning of the child’s fever in the mother’s caring relation to her child and examines the role of cultural background in the mothers’ experience.

## Methods

### Design

We chose a qualitative study design as data about the experiences of mothers caring for their children having a fever are lacking. A qualitative study using single in-depth interviews seemed the best setting to create an atmosphere in which mothers could talk about their experiences, emotions and possible culturally informed practices [26]. Grounded theory was chosen as analytical methodology as it is particularly suited to develop concepts that represent the participants’ experiences [27]. In order to avoid personal and cultural bias the research team comprised members from different professional and cultural backgrounds. In-depth interviews were performed from May through October 2008.

### Sampling and recruitment

In most families living in Germany the mother is the primary caregiver who stays at home, especially when children are younger than six years. Therefore, we restricted our sample to mothers in order to allow for comparisons [28]. As fever is most common in toddlers and young school children the mother’s youngest child had to be younger than eight years. Mothers were recruited while waiting with their feverish child for an appointment with a pediatrician. The German health care system allows parents to present their ill child to a pediatrician or GP working in private practice or to an emergency department of a children’s hospital. Especially in urban areas most children are regularly seen by pediatricians in private practice who perform vaccinations, preventive check-ups and provide acute outpatient care. (GPs however, are more involved in the care of children in more remote areas.) Mothers were approached by two co-authors (AS and MP) in the outpatient department of two pediatric hospitals and four practices of pediatricians in private practice in an urban region of the federal state of North Rhine-Westfalia, Germany. They explained the study to the mothers and asked for permission to call in two to four weeks after the initial contact - after assumed recovery of the child. At that point in time mothers were approached by telephone to explain the purpose of the study again and to ask whether they still agreed to participate; if so, an appointment was scheduled. Written consent was obtained before the actual interview started.

A mother was considered to have a Turkish cultural background if she or at least one of her parents was born in Turkey. A mother was considered to have a German background if she and her parents were born in Germany. This definition introduced by the Federal Office of Statistics encompasses mothers with very different migration and thereby acculturation experiences [29].

The role of a mother in a modern society can vary and is influenced by several contextual factors. In order to cover a broad range of experiences made by mothers we applied the principle of purposive sampling and interviewed individuals who showed differences in the following characteristics: number of children, mother’s age, family constellation (one/two parent family, grandparents nearby/not accessible), educational level and occupational status. Further, we included aspects in the sampling process that represent differences in the migration experience: length of stay in Germany, place of birth (Turkey or Germany) and native language (German or Turkish).

Participating pediatricians (where the mothers were approached) were recruited in meetings of two quality circles in the catchment area of Witten/Herdecke University. Quality circles are regular regional meetings of pediatricians to discuss clinical topics, ways to improve quality of care, as well as new developments in politics and funding. The two hospitals are teaching hospitals of Witten/Herdecke University. This sampling strategy served to cover a broad spectrum of housing areas and socio-economic differences.

### Data collection

We used a topic guide for the in depth-interviews which was developed in an interdisciplinary group and pilot tested in two interviews (See Additional files 1, 2 and 3). The questions covered the following themes:

Experiences from the last episode of childhood fever,

Being a mother in general,

Family context,

Living in Germany (for the immigrant mothers) and the local area.

Interviews were conducted by two of the authors who received training prior to and selective supervision during the interviews (TL: male, German background, pediatrican and AS: female, Turkish background, nurse). Turkish mothers were either interviewed by the male and the female interviewer together or by the female interviewer alone. TL did not interview mothers with a Turkish background alone in order to avoid irritations due to the Muslimic tradition of some participants. Interviews lasted 45-90 minutes, were audiotaped and transcribed verbatim. Follow-up interviews were not scheduled. Three interviews were conducted in Turkish by AS. The audiotapes were first transcribed and later translated into German by trained interpreters.

### Data analysis

The transcripts were analyzed in a group in order to maintain a high level of intersubjectivity and to avoid personal bias. Members came from different professional (medicine, nursing, ethnology) and cultural backgrounds (German, Turkish), both genders, different family status with some having children [26].

**Figure 1:**
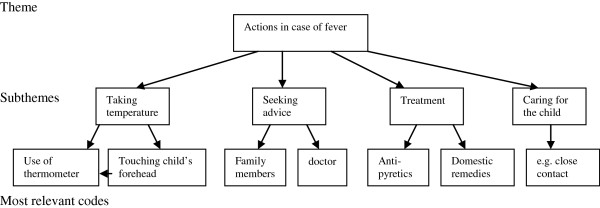
Illustration of the analytical process applied for the interviews.

Grounded Theory (Strauss & Corbin) was used as a framing methodology [27,30]. During the first phase of the analysis transcripts were analyzed by open coding. After the analysis of each transcript a detailed summary was written documenting the main themes, subthemes and most relevant codes. An example of this procedure is given in Figure 1. In the second phase open codes were grouped when they referred to similar themes, e.g. “family context”. In the third phase these categories were weighted regarding their relevance for the mothers’ experiences, and hypotheses regarding the relationship of the categories were formulated. In order to increase the generalizability of results, a social theory was searched that fitted the developed model best [31]. MAX QDA software was used to organize data.

### Ethics

The medical ethics committee of Witten/Herdecke University, Germany granted ethical approval (Reference 107/2007). All participants gave written consent to participate in the study and received no financial incentive.

## Results

### Participants

Initially 28 mothers were recruited. Eight either declined to be interviewed (6) or could not be reached by telephone (2). All agreed to be interviewed at their home, except for one, who wished to be interviewed at a hospital. The participants’ characteristics are shown in Table 1.

**Table 1:** Overview of participants

**No**	**Age**	**Family status**	**No of children (Age/yrs.)**	**Cultural background**	**Country of birth**	**Educational status**	**Vocational status**
1	27	Unmarried, with partner	1 (2)	Ger	Ger	c	Graduate public management
2	39	Married	3 (2, 4, 10)	Ger	Ger	b	Retailer
3	37	Married	1 (3)	Ger	Ger	c	Social worker
4	33	Married	1 (2)	Ger	Ger	c	Graduate in business administrator, at present housewife
5	33	Married	1 (9)	Ger	Ger	b	Nursery school teacher
6	33	Married	1 (4)	Turk	Ger	b	Administrative specialist
7	36	Married	3 (4, 10, 12)	Turk	Turk	a	Housewife
8	28	Married	2 (2, 4)	Turk	Ger	a	Waitress
9	36	Married	2 (2, 12)	Turk	Turk	a	Housewife
10	29	Married	2 (3, 5)	Ger	Ger	b	Office clerk
11	34	Divorced with partner	2 (1, 5)	Ger	Ger	b	Office clerk
12	42	Married	1 (7)	Ger	Ger	c	Marketing employee
13	37	Married	6 (7, 7, 15, 15, 16, 18)	Turk	Turk	a	Manual worker
14	28	Divorced, single	1 (6)	Turk	Ger	b	hairdresser
15	28	Unmarried without partner	2 (2, 12)	Ger	Ger	b	Housewife
16	38	Married	2 (7, 12)	Turk	Turk	a	Housewife
17	34	Married	3 (2, 5, 13, 15)	Turk	Turk	c	Housewife
18	30	Married	2 (4, 7)	Turk	Turk	b	Housewife
19	32	Divorced, single	1 (9)	Turk	Ger	c	Medical doctor
20	32	Married	1 (3)	Turk	Turk	b	Doctor’s assistant

Ger = German/Germany, Turk = Turkish/Turkey.

Educational status: a lower secondary education; b middle secondary education; c university entrance diploma.

### Findings

#### Fever as threat and the mother’s urge to protect the child

In most cases a fever develops rapidly and is associated with changes in the child’s appearance and behaviour. The child suddenly feels warm or even hot. Some mothers reported that their child becomes quiet, others that it becomes weepy and seeks her proximity. Sometimes, other signs of illnesses are recognized too, such as cough, sore throat, etc. The participants reported these changes precisely, indicating a high degree of attentiveness.

*“You could already notice that something wasn’t right. He also lay down on the couch all by himself, which he usually never does. And at some point, I just realized that he was getting a fever. It was actually really low at first, and then within a very short time, it went up to 40 by the evening. And he was listless. Well, he lay in the couch and it was as though I couldn’t really talk to him anymore, but he was also totally tired. And I just couldn’t figure it out:Now, is he listless or is he tired? “(Participant 9)*

However, fever means much more to the participants than an elevation of body temperature and a physiological event that can be described in signs, symptoms and a course of illness. The perceptions of the child’s changes seem to be instantly linked to an emotional response and to an impulse triggering an action. The emotional quality can be described as “concern” with different levels of intensity. Some mothers reported they could not sleep while their child has a fever. Others reported a more routine-like way to deal with this. The actions evoked by the child’s changes aimed to protect and comfort the child.

*“Well, when a child is actually sick, it’s horrible. Because when the child is sick, as a motherI am worried. Me, for example, I cannot sleep all night then. And afterwards, when I realize she’s a bit better now, then I, too, can also peacefully sleep a little. But otherwise, I have to watch out for her the whole time. If I somehow realize something is going on there, then I jump up immediately, because all my thoughts are only with her.” (Participant 14)*

As the observed changes are often impressive and the reason for the fever not obvious, it is experienced as a potential threat to the child’s wellbeing. This challenges the mother in her specific role and relationship to the child. There is a strong urge to protect the child from harm. The mother’s responsibility for the child is far-reaching and deeply rooted. It requires the mother to react upon the fever and “to do something”. Some mothers explicitly mentioned the fear of losing their child due to a disease.

“I think that’s what being a mother is all about. Well, I think that the relationship becomes closer. It has something to do with caring, but also wanting to help in the deepest sense of “preserving”, that is, one wants to somehow preserve the child’s life. And I think that it then doesn’t matter what he has, there is always a tremendous insecurity. Because the goal is, actually, that the child can grow up as well as possible. And then I think the bond gets even stronger.” (Participant 3)

While all participants expressed responsibility and concern for the child’s wellbeing initiated by the threat caused by fever, there were, however, differences in the areas of actions and explanations.

#### Explanations

Closely linked to the concern for the child’s wellbeing is the question concerning the cause of a fever. The explanations can be categorized thematically into four groups and are presented in Table 2. Although the groups of explanations seem to represent different ideas of pathogenesis we found them in combination within single interviews. Whereas “infection and transmission” and “climatic influences” were reported by mothers of both groups, we found accounts of the “supernatural causes” only interviews with Turkish mothers (in two instances). Interestingly, especially newborns up to the age of 40 days are seen to be particularly vulnerable to the evil eye and therefore often kept indoors.

**Table 2:** Explanations of the fever’s cause (Ger = German, Turk = Turkish)

**Explanation category**	**Causes**	**Predominant cultural background of mothers**
Infection and transmission	Infection from siblings	Ger & Turk
	Bacteria	Ger & Turk
	Viruses	Ger & Turk
	Infection in kindergarten	Ger & Turk
Climatic influences and temperature	Cold weather	Ger & Turk
	Inadequate, too light clothing	Ger & Turk
Supernatural causes	Evil gaze	Turk
Sign of other disease	Bronchitis	Ger & Turk
	Gastroenteritis	Ger & Turk
	Urinary tract infection	Ger & Turk
Others	Teething	Ger & Turk
	Overexertion of the child	Ger
	Emotional stress of the child	Ger

#### Actions and strategies

**Figure 2:**
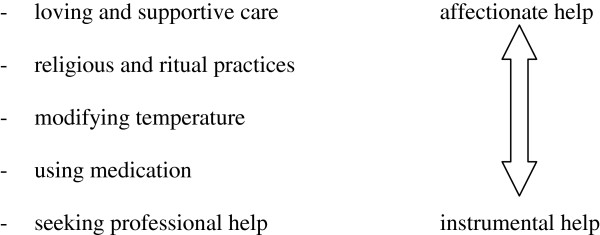
Types of strategies in response to fever.

We found a range of actions in response to the child’s fever that are summarized in Figure 2. Mothers chose different types of actions alone or in combination. They can be interpreted as lying on a continuum. On the one end there is affectionate and haptic care which represents a very close relationship to the sick child and serves to comfort the child. The measures on the other end have a stronger instrumental character. Giving medication and seeing a doctor are measures that imply an intervention in the physiology of the body or a prevention of serious harm through the doctor, respectively. They are primarily directed towards the disease and serve to protect the child from deterioration and harm.

All mothers reported on actions that cared for their child in a loving manner. This included supporting the child to rest or sleep, cuddling or reading stories. A frequent offering of drinks was also mentioned.

Praying for the child’s health was mentioned by mothers who normally pray. They included the sick child in their regular prayer. In our sample, some Turkish mothers were active Muslims who prayed five times a day. In contrast, none of the German mothers mentioned that their religious background played a role in caring for the child.

Two mothers mentioned the evil eye *(nazar)* as a possible cause of a child’s fever. Interestingly, especially newborns up to the age of 40 days are seen to be particularly vulnerable and therefore often kept indoors. These mothers reported on rituals to heal the child by burning salt and praying with a wise woman. The following quote illustrates the way evil eye causes harm the baby and what can be done to prevent and cure the child.

“It was my sister in law who gave her the evil eye. She had given birth to a child that had died 18 days later. And I guess she did it unintentionally; I don’t know, only God knows. After she had left the door the fever started rising. Afterwards my mother said it was her because she also did not say “Mashalla. That’s what we usually say when we look at a child to protect it. […] Often the evil eye causes fever and children can even die if you do not react promptly. […] My daughter had a fever for three days and nights. The suppositories given by the pediatrician did not help. My mother called for a wise woman. She put salt in the corners of the room and burnt the rest on the hotplate. Then she said something and I had to repeat it. Afterwards the fever was gone.” (Participant 17)

Interestingly, modifying the child’s temperature included measures of both lowering and elevating. Lowering the temperature was attempted by light clothing, the breathing of cool air, cool wraps, sponging with water, alcohol or vinegar and the use of antipyretic drugs. The use of additional blankets and warm clothing was used to elevate the child’s temperature.

We found sponging with vinegar and attempts to elevate temperature in some Turkish mothers. The other measures were mentioned in both groups.

The use of antipyretics plays an important role in most mothers’ fever management. The temperature above which paracetamol or ibuprofen was given varied between 38 and 40°C. Only German mothers mentioned the use of homeopathic and naturopathic medication.

Whereas mothers tried to treat and help the child with self-administered measures, they were also anxious about not recognizing a serious condition. The doctor was therefore supposed to assess the child’s condition.

#### Role of the mother’s cultural background

In this study we applied an understanding of culture as values, beliefs and practices shared by a group of individuals [26]. Thus, it comprises observable activities and underlying symbols and meanings [32]. A relation to a mother’s cultural background was assumed when mothers drew on their family tradition or provenience.

Whereas the theme “concern and responsibility” was the central category in the interviews shared by all mothers, the areas of explanations and strategies are influenced by the mothers’ cultural backgrounds. Therefore, the quality of the cultural background’s influence could be described as “tinging” these factors instead of being an independent factor in its own right.

#### Relation of findings to theory – the caregiving system model

The central theme in the mothers’ accounts were “concern and responsibility” for the child. In a situation of uncertainty and possible threat for the child caused by fever, the mothers experienced an urge to protect the child from harm. The perception of the child’s changes, the mother’s emotional response and the perceived need to react upon the fever are closely linked to each other and situated in the relationship of the mother to *her* child. The explanation and action a mother chooses depends on her personal history, cultural background and opportunities at hand.

These findings correspond well with the caregiving system model developed by George and Solomon [33,34]. Following the basic attachment theory model proposed by Bowlby [35], the authors emphasized that a parent’s contribution to his or her relationship with the child is guided by a biologically based behavioural system called the caregiving system. The parent’s caregiving behavioural system and the child’s attachment system are reciprocally coordinated to achieve the goal of proximity and protection from harm. To this end, the parent naturally pays attention to specific cues from the child that he or she needs care (e.g., crying, signalling the need to be close) and evaluates the degree to which the parent believes the larger context is dangerous for the child (i.e., the degree to which the parent sees the situation as life threatening).

The term behavioural system implies that several different actions are possible to achieve the same outcome. That is, depending on the age of the child, the parent’s evaluation of the child and context, and other factors including cultural practices, parents will show a range of different responses and behavioural strategies to achieve this goal. What these responses have in common is that they are “driven” by the fundamental need to protect their child from harm.

With respect to the emotional quality of care giving Solomon and George state that “mothers express intense feelings of pleasure and satisfaction when they are able to protect and comfort their children and that they experience heightened anger, sadness, anxiety or despair […] when their ability to protect and comfort the children is threatened or blocked” (page 835).

There are several aspects of the findings in our study that fit particularly well into the caregiving system model.

1. The goal to protect the child and maintain its life was a powerful motivation for the mothers interviewed in this study and represents a central element in the caregiving system model.

2. A high vigilance towards the child’s state and the evaluation of the threat in order to decide what to do is an important finding in this study and a specific element of the activated care giving system.

3. A strong emotional participation was found in the participant’s accounts and is also typical of an activated care giving system.

4. Influence of contextual factors: The goal of the care giving system is the protection of the child. However, a range of actions can serve to achieve this goal depending on the mother’s resources and other contextual factors. Thus, the care giving system model is specific towards its behavioral goal and offers a space for different behaviours and strategies at the same time.

5. Care giving behaviour is dynamic and individual with regard to a child over time. The mothers in this study also reported the experience of change over time or by number of siblings.

## Discussion

In this qualitative study, we analyzed the experiences of mothers when their child has a fever. The mothers’ perceived changes of the child’s state are closely linked to an emotional response characterized as “concern” and a deeply rooted urge to protect her child from harm. On this level of analysis we did not find any differences between mothers with a German background and those from a Turkish background. We found the caregiving system model by Solomon and George to offer a well-fitting theoretical perspective for the mothers’ reported experiences. The actions and strategies taken by mothers varied and can be characterized as lying on a continuum between affectionate and instrumental care and found the cultural background to influence the explanations of fever and the selection of actions.

As in many other studies addressing the experience of patients and family members, a qualitative approach was used. The sampling criteria were chosen to capture a broad spectrum of experiences and formulated on the basis of existing literature and our assumptions outlined above. In order to avoid personal or professional bias in the interpretation of data, the analysis team was multiprofessional and multicultural. Using social theory in the interpretation of the results helps to extrapolate our findings to other settings or situations that could activate the care giving system, e.g. other childhood illnesses or pain [36].

In addition to a number of studies on parent’s views on fever that predominantly focused on parents’ knowledge of fever, we showed in our study that fever is very much a challenge for the mother in her unique relation to the child. Whereas the parents’ view of fever has often been described as being irrational [4,14], we found the participants’ accounts to represent a rational approach when considering their responsibility, experienced uncertainty and perceived options for actions at hand.

This main finding corresponds well with a qualitative study carried out by Kai who showed similar results in a qualitative study analyzing parents’ worry when their children are acutely ill [15]. He found that parents’ concerns were expressed within the context of keenly felt pressure, emphasizing parents’ responsibility to protect their child from harm. Parents’ sense of personal control and the perceived threat posed by an illness were found to be the main factors influencing their experience. In addition to these findings, our study integrates the mothers’ experience into the context of caregiving and attachment, a field that has rapidly developed during the past 20 years. This area of developmental psychology offers a promising theoretical perspective to better understand mothers’ concerns and behaviours when their children are ill.

From a clinical perspective some of the reported treatment strategies are relevant to physicians. Modifying the child’s body temperature was an important goal for some participants and it was aimed for by different means: cool wraps, sponging with water, vinegar and alcohol and administration of antipyretic drugs. The guidelines by the American Academy of Pediatrics and the National Institute for Clinical Excellence (NICE) do not recommend sponging to reduce temperature. Instead, “the use of antipyretic agents should be considered in children with fever who appear distressed or unwell. Antipyretic agents should not routinely be used with the sole aim of reducing body temperature in children with fever who are otherwise well. The views and wishes of parents and carers should be taken into consideration.” [37,38].

In this study we found the “evil eye” to be a particularly interesting explanatory model of fever as it can hardly be linked to the scientific model of infection commonly used to explain fever. However, from an anthropological perspective it is a common finding that other people are blamed for one’s ill health. Thus, the “evil eye” reported by two mothers with a Turkish background is probably not specific for the Turkish culture. Helman states that ““evil eye” as a cause of illness has been reported in different European countries, the Middle East and North Africa with different names, e.g. “mal de ojo” in Hispanic cultures and “ayin ha-rah” in Hebrew”. The concept of the “evil eye” points to a social aetiology of illness as it relates to the fear of envy in the eye of the beholder. “The possessor of the evil eye usually harms unintentionally, is often unaware of his or her powers and is unable control them” [26].

A better understanding of the experience of mothers caring for an acutely ill child could help to improve the communication between physician and parents. Deeper insights in the relationship of illness experience and cultural background may help address the concerns of parents more effectively. This could improve counselling of parents and eventually empower them to take care of their child more independently and thus reduce the use of emergency services. Further, a better insight into the views and theories of parents could help to offer specific information about warning signs of serious conditions and thereby help to prevent delay of treatment. Based on the findings of this study we argue that the emotional intensity of concern expressed by the participants of this study and the urge to protect the child from harm needs to be acknowledged by the practitioner. In order to enable parents to care for their feverish child effectively doctors should provide clear information and advice.

This study has several limitations. We interviewed mothers with a German or Turkish background living in Germany. Thus, one has to be cautious in generalizing our findings to other populations, e.g. mothers with a Turkish background living in Turkey or mothers from other countries. Further, fathers were not interviewed limiting the generalizablity to all parents. With regard to the theory of caregiving we used this theory on its conceptual level. Clinical applications used to diagnose disorganized caregiving as a cause of attachment problems were not used in this study. We interviewed mothers of children aged younger than 8 years. As the relationship between mothers and children changes remarkably during adolescence an extrapolation of our findings to later developmental stages does not seem warranted.

## Conclusion

To mothers childhood fever means much more than just an elevated temperature. By applying the caregiving system model maternal actions can be understood as an understandable attempt to protect the child from harm. In order to test the applicability of this approach on other scenarios we intend to explore the experiences of parents in other situations that put children at a certain risk, e.g. pain, operations, obesity etc.

Childhood fever is a common reason for consulting a primary care physician as it causes concern and uncertainty. When counselling parents, physicians should acknowledge the mothers’ caring role and emotional state and provide clear and specific advice that helps the parent to better control the situation at home. Further, physicians should actively inquire about actions taken at home as some of them carry the risk of doing harm to the child even if this was not intended. Moreover, eliciting parents’ explanations and treatment strategies seems important for a trustful relationship and for effective counselling in order to avoid harm to the child. The activated care giving system could provide a resource for an effective collaboration between parent and physician in the child’s interest.

## Competing interests

TL was supported by a grant of the Federal Ministry of Education and Research, Germany (01GK0716). The authors declare that they have no competing interests.

## Authors’ contributions

TL was the principal investigator of the study. He participated in the conception of the study, the development of the interview guide, data collection and data analysis. He took the lead on drafting the manuscript. MP participated in the conception of the study and the data analysis. AP participated in the data collection and conducted all interviews in Turkish. She also participated in the data analysis and provided valuable insights into cross-cultural commonalities and differences. VP, SW and WS supported TL and AS during the process of data collection by providing supervision and feedback. They also participated in the analysis of data. All authors read and approved the final manuscript.

## Pre-publication history

The pre-publication history for this paper can be accessed here:

http://www.biomedcentral.com/1471-2296/14/35/prepub

## Supplementary Material

Additional file 1 Interview Topicguide - English version. Click here for file

Additional file 2 Interview Topicguide - German version. Click here for file

Additional file 3 Interview Topicguide - Turkish version. Click here for file
