# Supplementary material for: Activation of the maternal caregiving system by childhood fever – a qualitative study of the experiences made by mothers with a German or a Turkish background in the care of their children
Source: BMC Fam Pract. 2013 Mar 18;14:35. doi: 10.1186/1471-2296-14-35 (PMC3607993; doi:10.1186/1471-2296-14-35)
Supplement: Additional file 1 — Interview Topicguide - English version. [file 1471-2296-14-35-S1.doc]

**Topic Guide**

**Experiences made by mothers with a German or Turkish background in childhood fever**

After welcome, introduction, providing information on study and obtaining informed consent the interview starts.

**Part 1 – Fever and Illness**

1. Could you please tell how it was like when your child had a fever the last time?
2. How was your child?
3. How did you feel?
4. What do you think caused the fever?
5. Do you have any other ideas what could have caused the fever?
6. What is fever in your opinion?
7. Why do you think got your child sick?
8. What is the most dangerous thing that could have happened to your child?
9. What actions did you take?
10. If a doctor was seen: What did the doctor say? Was he/she helpful? Why?
11. Was there a fever episode in the past with which you dealt differently?
12. Could you please tell me what happened at that time?
13. What did you do at that time? (again questions 3-6)
14. Do you apply natural therapies or treatment strategies used in Turkey when your child is unwell?
15. When someone is ill in your family who is commonly asked first?
16. Did you observe any positive effects on your child associated with the fever?

For mothers born in Turkey

1. At present, do you deal with diseases in a different way than you know it from Turkey?
2. Where do you see commonalities?
3. Where do you see differences?

**Part 2 – Role of the mother of a feverish child**

1. What does it mean for you to be a mother?
2. What does it mean for you when your child is unwell?
3. How would you describe your role as a mother when your child is ill?

**Part 3 – Illness in the family**

1. Do you know a child among your family and friends with a chronic illness or impairment? If so, could tell me about it? What does it mean to you?
2. Who is part of your family?

**Part 4 – living in Germany (for mothers with a Turkish background)**

1. When you talk to friends and family members about life in Germany, where do you see the main differences?
2. When you talk to Germans and look at their lifes, where do you see the main differences to your life?
3. Talking about caring for children, where do you see differences to German families?
4. With regard to medical care for children what are the main differences between Turkey and Germany?
5. How do experience the medical care for your children?
