# Supplementary material for: Activation of the maternal caregiving system by childhood fever – a qualitative study of the experiences made by mothers with a German or a Turkish background in the care of their children
Source: BMC Fam Pract. 2013 Mar 18;14:35. doi: 10.1186/1471-2296-14-35 (PMC3607993; doi:10.1186/1471-2296-14-35)
Supplement: Additional file 2 — Interview Topicguide - German version. [file 1471-2296-14-35-S2.doc]

**Interviewleitfaden zum Thema**

**„Fieber im Kindesalter in deutschen und türkischen Familien“**

**Einleitung**

Danke fürs Kommen und die Möglichkeit des Interviews. Hintergrund der Untersuchung (Bedeutung der elterlichen Pflege des Kindes und Interesse an den Lebensumständen in Deutschland)

Füllen Sie zunächst bitte den Fragebogen aus (Angaben zu Alter, Anzahl der Kinder, Geburtsland etc)

**Teil 1 (Vorgehen beim fiebernden Kind)**

1. Könnten Sie erzählen wann ihr Kind (bei mehreren das jüngste) zuletzt Fieber hatte?
2. Wie war das? Erzählen Sie bitte wie es ihrem Kind dabei ging?
3. Was glauben Sie, hat das Fieber verursacht?
4. Haben Sie noch andere Ideen, wodurch das Fieber hätte hervorgerufen werden können?
5. Was ist eigentlich Fieber? Wie entsteht das Fieber?
6. Warum glauben Sie, ist ihr Kind gerade jetzt krank geworden?
7. Warum glauben Sie, ist gerade Ihr Kind krank geworden?
8. Was glauben Sie war das Gefährlichste, was ihrem Kind hätte passieren können?
9. Was haben Sie unternommen? Beispiele
   - - Behandlung des Kindes (allgemein, Anwendungen, Präparate, Medikamente)
     - Innerhalb der Familie (sich besprechen, Rat einholen)
     - Sind sie zum Arzt gegangen? Was hat sie bewegt, zum Arzt zu gehen?
     - Falls ein Arztbesuch stattfand: Was hat der Arzt ihnen gesagt? Waren Sie mit dem Besuch zufrieden? Wenn ja, warum? Wenn nein, warum nicht?
10. Gab es in der Vergangenheit eine Krankheit ihres Kindes mit Fieber, wo sie sich ganz anders verhalten haben?
11. Können Sie erzählen, was damals passiert ist?
12. Was haben sie damals unternommen? (Wiederholung der Fragen 3-6)
13. Verwenden Sie Naturheilverfahren oder andere Behandlungsweisen, z.B. aus der Türkei, wenn es ihrem Kind nicht gut geht?
14. Wenn bei Ihnen in der Familie jemand erkrankt, wer wird als erstes um Rat gefragt, was zu tun ist?
15. Hat die Krankheit des Kindes im Nachhinein auch etwas Gutes für das Kind bewirkt?
16. Welche anderen Krankheiten kennen Sie, bei denen Fieber auftritt?

Für türkische Familien, wenn Eltern in der Türkei geboren sind:

1. Gehen Sie heute mit Krankheiten ihrer Kinder anders um, als Sie das von sich aus der Türkei kennen?
2. Was war gleich, was war anders? Können Sie das beschreiben?
   - - Krankheiten
     - Behandlungsformen
     - Vorgehen in der Familie
     - Aufsuchen von ärztlicher Hilfe

**Teil 2 (Rolle als Mutter des kranken Kindes)**

1. Was bedeutet es für Sie Mutter zu sein?
2. Was bedeutet es für Sie als Mutter, wenn Ihr Kind krank ist?
3. Wie würden Sie Ihre Aufgabe als Mutter beschreiben, wenn Ihr Kind krank ist?

**Teil 3 (Krankheit in der Familie und im Bekanntenkreis)**

1. Gibt es in ihrem Umfeld ein Kind mit einer chronischen Erkrankung oder einer Behinderung? Könnten Sie bitte berichten, wie sie das erleben?
2. Wie sind sie in die Betreuung dieses Kindes eingebunden?
3. Wer gehört bei Ihnen alles zur Familie? (in Hinblick auf die Pflege eines kranken Kindes und bei offiziellen Festen, wie z.B. der Beschneidung?)

**Teil 3 (Leben in Deutschland)**

Ich hätte nun ein paar Fragen an Sie dazu, wie es ist als Mutter (und Frau) in Deutschland zu leben.

1. Wenn Sie mit Angehörigen reden, die in der Türkei leben, was ist Ihr Eindruck, worin sich das Leben in Deutschland am meisten unterscheidet?
2. Wenn Sie mit Deutschen reden oder deren Leben beurteilen, wo sehen Sie die stärksten Unterschiede zu ihrem Leben?
3. Wenn es um die Betreuung Ihrer Kinder geht, wo liegen hier die Unterschiede zum Leben in der Türkei?
4. Wenn es um die Betreuung der Kinder geht, wo sehen Sie Unterschiede zu den deutschen Familien?
5. In Hinblick auf die medizinische Versorgung ihrer Kinder, wo liegen da die Unterschiede zum Leben Ihrer Angehörigen in der Türkei?
6. Wie empfinden Sie die Versorgung Ihrer Kinder? Gut? Was könnte besser sein?

**Teil 4 (Nachfragen, außerdem)**

1. Verwenden Sie manchmal das blaue Auge?
2. Gibt es in ihrer Gegend eine Gruppe der Dertlesmek? Haben Sie schon einmal Kontakt zu den Dertlesmek gehabt?
3. In welchen Situationen suchen Sie den Hoca auf?
