# Supplementary material for: Activation of the maternal caregiving system by childhood fever – a qualitative study of the experiences made by mothers with a German or a Turkish background in the care of their children
Source: BMC Fam Pract. 2013 Mar 18;14:35. doi: 10.1186/1471-2296-14-35 (PMC3607993; doi:10.1186/1471-2296-14-35)
Supplement: Additional file 3 — Interview Topicguide - Turkish version. [file 1471-2296-14-35-S3.rtf]

"Alman ve Türk ailelerde cocugun ateslenmesi"


1. Bölüm (Cocugun ateslenmesi durumunda yapilanlar)

1) En son ne zaman  cocugunuzun ateslendigini (birkac cocuk varsa en kücügü) bize söyleyebilirmisiniz?
Bu olayi nasil yasadiginizi aciklarmisiniz?

2) Olayi biraz daha aciklarmisiniz, cocugunuzun durumu o zaman nasildi?

3) Sizce cocgun atesinin cikmasina ne sebep olmustu?

4) Sizce atesin cikmasina baska ne sebep olmus olabilir?

5) Ates nedir ? Ates nasil cikar?

6) Sizce neden cocugunuz simdi hasta oldu?

7) sizce neden özellikle sizin cocugunuz hasta oldu?

8) Sizce bu cocugunuzun basina gelebilecek ne tehlikeli seymiydi?

9) Cocugunuz hastalaninca ne yaptiniz? örnekler
- cocugun tedavisi (genel tedavi, uygulamalar, ilaclar)
- aile ici (akil alma, birbirine danisma)
- doktora mi gittiniz ? doktora gitmenize sebep olan neydi?
- Eger doktora gittiyseniz, doktor size ne dedi? Doktora gittiginize memnun oldunuz mu? evet se neden, hayir ise neden memnun kalmadiniz?

10) Gecmiste  cocugunuzun atesi ciktiginda tamamen farkli davrandiginiz bir durum oldu mu?

11) Bize gecmiste olan olayi anlatirmisiniz?

12) O zaman ne yapmistiniz? (3-6 sorularinin tekrari)

13) Cocugunuz iyi olmadiginda dogal bitkilerle, alternatif, yada türkiyeden ögrendiginiz baska yöntemlerle cocugunuzu tedavi ediyormusunuz?

14) Ailenizden biri hastalandiginda önce kime akil danisilir, kime ne yapilacagi sorulur?

15) Cocugunuzun hastaligi daha sonradan ona faydali birsey sagladi mi?

16) Baska insanda ates cikmasina sebep olan hangi hastaliklari taniyorsunuz?


Ebeveynleri Türkiyede dogmus olan aileler icin

17) Bugün cocugunuz hastalandiginda artik eskiden Türkiyeden ögrendiklerinizden farkli bir sekilde davraniyormusunuz?

18) ayni olan seyleri ve farkli olan seyleri siralarmisiniz ? bunlari aciklatmisiniz?
 - Hastaliklar
- tedavi yöntemleri
- ailedeki davranis ve uygulama sekli
- doktora gitmek ve yardim almak


2. Bölüm (Hasta cocuga Anne rolünde olma)

19) Sizin icin Anne olmak ne demektir?

20) Anne olarak cocugunuzun hastalanmasi sizin icin ne demektir, ne ifade ediyor?

21) Anne olarak sizce cocugunuz hastalandiginda görevleriniz nelerdir?


3. Bölüm (Aile ve tanidik cevresinde hastalik)

22) Sizin etrafinizda, cevrenizde kronik hastaligi olan yada sakat olan bir cocuk var mi? Varsa bunu nasil yasadiginizi bize anlatirmisiniz?

23) Bu cocugun bakimiyla ne sekilde ilgileniyorsunuz, sorumluluklarinizi anlatirmisiniz?

24) Siz de aileye ait olanlar kimlerdir. (Hasta cocugun bakimi, resmi tatiller ve sünnet gibi merasiymlerde)


3. Bölüm (Almanya da yasamak)

 Bir kadin ve anne olarak almanya da yasamakla ilgili birkac sorum olacak.

25) Türkiye de yasayan akrabalarinizla konustugunuzda, görüstügünüzde,gördügünüz  Almanyadaki yasamin Türkiden farkli olan en önemli yönü sizce nedir?

26) Bir Almanla konustugunuzda yada onun hayatiyla ilgili yorum yaptiginizda, sizce onun sizin hayatinizdan en fazla farkli olan yönleri nelerdir?

27) Sözkonusu olan cocuk bakimi olunca sizce burdaki hayat ile türkiyedeki arasinda ne gibi farklar var?

28) Sözkonusu olan cocuk bakimi olunca sizce alman aileleri ile aranizdaki farklar nelerdir?

29) Cocuk tedavisi ve tibbi bakimi alaninda Türkiyedeki akrabalarinizla aranizda olan farkliliklar nelerdir?

30) Cocugunuzun bakimi ve tedavisi ile alakali sartlari nasil buluyorsunuz? iyi mi? ne daha iyi olabilirdi?

31) Nazamr boncugunu bazen kullaniyormusunuz?

32) Cevrenizde dertlestiginiz bir grup var mi?  Daha önce dertlesme gruplariyla iliskiye gectiginiz oldu mu?

33) Hangi durumlarda bir hoca ziyaretini gerekli bulursunuz?
